# When caring hurts: Chronic back pain, anxiety, and depression among caregivers of chronically ill children in Nigeria

**DOI:** 10.1371/journal.pmen.0000402

**Published:** 2025-08-12

**Authors:** Adefunke DadeMatthews, Temitope Ogundare, Oluwagbemiga DadeMatthews, Sewanu Awhangansi, Teanna Moore, Samsudeen Opeyemi Alao

**Affiliations:** 1 Department of Psychiatry, National Postgraduate Medical College, Lagos, Nigeria; 2 Department of Psychiatry, Boston Medical Center, Boston, Massachusetts, United States of America; 3 Department of Psychiatry, Boston University School of Medicine, Boston, Massachusetts, United States of America; 4 School of Kinesiology, Louisiana State University, Baton Rouge, Louisiana, United States of America; 5 Leicestershire Partnership NHS Trust, Leicestershire, United Kingdom; 6 Accessible Teaching, Learning, and Assessment Systems (ATLAS), University of Kansas, Lawrence, Kansas, United States of America; 7 Department of Neonatology, Leicester Royal Infirmary, Leicester, United Kingdom; Manipal Academy of Higher Education, INDIA

## Abstract

Children with chronic health conditions need substantial care to address all aspects of their health and recovery. Parents (caregivers) often shoulder significant financial, physical, social, and emotional responsibilities in meeting these needs. When access to vital resources such as information, financial support, and respite care is limited, parents’ physical and emotional well-being can suffer, exacerbating the stress associated with caregiving. This study aims to determine the prevalence and correlates of chronic back pain, depression, and anxiety in caregivers of children with chronic diseases in three tertiary hospitals in Nigeria and the associations with burden of care. A cross-sectional study of caregivers of children under 18 years with chronic medical conditions from three tertiary hospitals in Nigeria was conducted. A sociodemographic questionnaire, Patient Health Questionnaire 9 (PHQ-9), Hamilton Anxiety Rating Scale (HARS), Zarit Burden Interview (ZBI), and the Oswestry Disability Index (ODI) were administered. Principal component analysis, multiple linear regression, and mediation analyses were conducted. 184 caregivers, mostly females (84%) and mothers (80%) participated. Mean age for this group was approximately 37 years-old (SD = 7.98). Results from multiple regression analyses indicated that the total burden of care predicted higher levels of depression (*p* < 0.01, 95% *CI* = [0.32, 0.74]) and higher levels of anxiety (*p* = 0.02, 95% *CI* = [0.09, 1.03]). Findings from the mediation analyses indicated that the burden of care did not mediate the relationship between back pain and depression (*p* = 0.13) and back pain and anxiety (**p* *= 0.53). This study highlights the high prevalence of anxiety, depression, and chronic back pain among caregivers of children with chronic medical conditions in Nigeria. Caregiver burden and chronic back pain are also associated with anxiety and depression.

## Introduction

Children with chronic diseases require substantial support and care that addresses various aspects of their health and recovery. This includes not only rehabilitation services tailored to their specific medical needs but also comprehensive support for their physical, social, and psychological well-being. The bulk of the long-term and short-term financial, physical, social, and emotional responsibilities related to children’s healthcare needs primarily rest on caregivers [1,2]. The burden is weightier for caregivers in poorly resourced settings like Africa, where essential amenities like chairs, clean water, and waiting rooms are usually unavailable [3,4]. Parents caring for chronically ill children have their physical and emotional well-being detrimentally impacted by the absence of vital resources like access to information, financial support, and respite care [5,6]. Research should prioritize understanding the mental and physical distress that caregivers experience while providing care for children.

High levels of psychological discomfort, such as depression, are linked to the tremendous physical strain that caregivers experience [7]. Caregivers who experience mental or emotional strain have a higher mortality rate compared with those without strain or non-caregivers. Psychological distress can negatively affect the caregiver’s and hospitalized child’s quality of life [8].

Caregivers of children with chronic conditions frequently suffer from anxiety and depression [7,9]. Anxiety is associated with the severity of patient symptoms, a more significant impact on caregivers’ itineraries, greater patient depression, and worse caregiver social functioning [10]. Caregiver psychosocial problems influence the physical health of the chronically ill child and the psychosocial functioning of the caregiver. For instance, maternal depression affects the child’s adherence to therapy [11]. Therefore, interventions to control anxiety and depression among caregivers are important for the patient’s and the caregivers’ health [12]. Given the unique challenges of the Nigerian healthcare system, the inadequate or non-existent facilities for caregivers’ comfort, such as waiting areas with chairs, bedside chairs, restroom facilities, and water stations, many are forced to stand for long periods or sit/sleep in uncomfortable positions on the floor. The lack of seating and extended standing or floor sitting increases the likelihood of chronic back pain.

Chronic back pain is commonly observed among female caregivers of children with physical disabilities [13]. Back discomfort that persists over time is linked to higher psychological distress [14]. The interaction between the physical and mental strain experienced when caring for a hospitalized child is one reason why there is a link between chronic back pain and increased psychological distress among caregivers, particularly female caregivers of children with chronic illnesses [15,16].

Like other countries, Nigeria has individual risk factors (e.g., gender, history of traumatic life events and stressful situations in childhood, and individual ability to manage stress/ resilience) for psychological distress [17,18]. Additionally, community risk factors (e.g., lack of social support, stigma from community members, and the spiritualization of illness) contribute to psychological distress [19–21]. Further, societal risk factors (lack of health insurance, lack of support from employers, lack of government support with funds in cases of low socioeconomic status) exist and add to the likelihood of developing psychological distress [22–25].

Despite the prevailing risk factors for psychological problems, there is a shortage of research into the prevalence of anxiety, depression, and chronic back pain among caregivers of children with chronic disorders. There is also insufficient information on the relationship between the burden of care and psychological distress among this population.

This study aims to investigate the prevalence and correlates of chronic back pain, depression, and anxiety in primary caretakers of children with chronic diseases in three tertiary hospitals in Nigeria and to determine the associations between these variables and their relationship with the burden of care.

### Research questions

1. What are the prevalence and correlates of anxiety, depression, and chronic back pain among caregivers of children with long-term illnesses in tertiary hospitals in Abeokuta, Nigeria?
2. What are the associations between sociodemographic factors, caregiver depression, anxiety, chronic back pain, and burden of care among caregivers of children with chronic conditions in tertiary hospitals in Abeokuta, Nigeria?
3. Do chronic back pain and burden of care predict depression among caregivers of children with chronic conditions in tertiary hospitals in Abeokuta, Nigeria?
4. Do chronic back pain and burden of care predict anxiety among caregivers of children with long-term illnesses in tertiary hospitals in Abeokuta, Nigeria?
5. Does caregiver burden mediate the relationship between chronic back pain and depression?

### Hypothesis

Our study centers on three hypotheses. First, we believe that caregivers with a higher burden of care will have higher depression and anxiety scores. In addition, we expect chronic back pain will predict depression and anxiety. Second, the caregiver burden will mediate the relationship between chronic back pain and depression. Last, we expect to find associations between elements of socio-demography (e.g., sex, age, and marital status), depression, anxiety, chronic back pain, and burden of care.

## Method

### Ethics statement

The Federal Medical Center, Health Research Ethics Committee (FMCA/470/HREC/01/2021/07), Sacred Heart Hospital Ethical Committee (SHH/EC/EA/09/06/21) and the Research Ethics Committee of State Hospital, Nigeria (SHA/RES/VOL4/226) gave ethical clearance and permission for the study. A signed and written permission was obtained from participants after they were told the study’s nature and objectives. Anonymity and confidentiality were upheld for all collected data.

### Study design

We used a cross-sectional research design to explore the relationship of the previously listed factors with respect to the caregivers of children with chronic medical conditions receiving care at three tertiary hospitals in Nigeria. This study represents the first part of a larger study.

### Participants

The study focused on caregivers, aged 18 or older, spoke English or Yoruba, had a history of anxiety, depression, persistent back pain, or other chronic medical disorders caring for children with a chronic condition a minimum of four hours a day for the past six months and are involved in helping with activities of daily living. Caregivers consented to participating in the study. We only selected caregivers who cared for children younger than age 18 with a chronic medical condition (e.g., cancer, chronic kidney disease, congenital heart defects, sickle cell disease), experienced an injury (e.g., burns or fractures) that resulted in a hospitalization lasting one month or more, or undergone surgical conditions such as Hirschsprung’s disease or other conditions that require long-term surgical treatment. We excluded those caring for children with intellectual disabilities or psychiatric illnesses and those caring for an adult with a chronic medical condition.

### Procedure

Case records of patients at the outpatient clinics and hospital inpatient unit were reviewed to determine who could be a candidate for participation in our study. We recruited participants using convenience sampling. We explained the study’s purpose to participants and stated that their participation was voluntary, would not affect the quality of care, and that, they were free to leave the study whenever they wanted without facing any repercussions. Once we received consent, we administered hard copy questionnaires to selected participants.

Questionnaires were administered from 10/06/2021 to 09/05/2022, and presented in either Yoruba or English, given these languages are primarily spoken in southwest Nigeria, where our study was conducted. Yoruba versions of the questionnaires were generated using iterative back-translation provided by professional linguists and clinicians versed in English and Yoruba. To ensure confidentiality of their responses, participants completed the questionnaires in a private area. We also deidentified the data by assigning a serial number to each participant to ensure participants’ anonymity.

### Measures

**Sociodemographic questionnaire:** Interviewers administered a sociodemographic questionnaire created by the authors to collect sociodemographic data on age, gender, employment, educational attainment, expected monthly income/allowance, and source of finance for medical care. The second part included the patient’s age, gender, and health-related information, such as the kind of chronic condition, the frequency of emergency room (ER) visits per year, the length of the hospitalization, and the inpatient ward.

**The Patient Health Questionnaire-9** [PHQ-9; 26]: It is a self-administered depression scale assessing the 9 DSM-IV criteria for major depression. Each question is given a score ranging from 0 (not at all) to 3 (almost every day). The lowest overall score is zero and the highest is 27. Scores over 10 are indicative of major depression. In primary care research, the PHQ-9 demonstrated strong internal reliability with a Cronbach’s alpha of 0.86 and test-retest reliability of 0.84 [26]. It has high psychometric qualities and has been utilized in numerous Nigerian studies and demonstrated good psychometric properties [27–29]. Test-retest reliability in the study was 0.89, while the internal consistency of the PHQ-9’s items, as measured by Cronbach’s alpha, was 0.85 [28].

**The Hamilton Anxiety Rating Scale** [HARS; 30]: The 14-item Hamilton Anxiety Rating Scale, developed in 1959, is a clinical interview tool for assessing anxiety symptoms. The scale evaluates a wide variety of signs and symptoms that are present in all eight DSM IV anxiety disorders. The generalized anxiety disorder (GAD) severity test is most frequently used for this purpose. According to Shear et al. (2001), the reliability (test-retest) and Cronbach’s alpha (internal consistency) for GAD were both 0.79 (0.66-0.87) and 0.82, respectively. With a total score of 56, individual items are assessed on a scale from 0 (not present) to 4 (severe). Scores of 7 and below denotes no anxiety, between 8 and 14 denote mild anxiety, between 15 and 23 indicate moderate anxiety, and 24 or higher, severe anxiety [31]. Many studies conducted in Nigeria have employed HARS [32–35].

**Zarit Burden Interview** [ZBI; 36]: It is a 22-item questionnaire to gauge how burdensome the caregiver considers family caregiving. A total score is calculated by adding the item scores, ranging from 0 to 88, with higher scores signifying a heavier burden. The 22 items are evaluated using a 5-point Likert scale, where 4 represents “nearly always,” and 0 represents “never.” The questions center on the caregiver’s mental and physical health, financial situation, social life, and interaction with the patient. ZBI showed good reliability and validity for measuring caregiver burden. With 149 people investigated, the test-retest reliability’s intraclass correlation was 0.89, and Cronbach’s alpha was 0.93 [37]. With a strong Cronbach’s alpha of 0.90 and a split-half correlation value of 0.84, ZBI has been utilized in Nigerian research with good internal consistency [38–41]. ZBI has a 70% sensitivity and a 68.4% specificity, respectively [39].

**The Oswestry Disability Index** [ODI; 42] was developed to evaluate functional impairment from chronic conditions, particularly chronic back pain. The ODI is a common measure of disability that asks patients with chronic back pain about their tolerance levels for ten different activities, including standing, sitting, walking, lifting, self-nurturing, having sex, and traveling. Each item contains six statements (scored from 0-5) reflecting their increasing or decreasing tolerance for the activity. A percentage is used to represent the overall score. Fairbank et al., 1980 discovered 0.99 test-retest reliability on two daily and consecutive measurements and provided some evidence of validity by showing that treated patients’ scores declined as their mobility increased [42]. Numerous Nigerian studies have employed the ODI [43–46]. The Yoruba ODI’s internal consistency was 0.97 (Cronbach’s alpha), and the intraclass correlation coefficient produced by inter-rater reliability was 0.93 [44].

### Data analysis

Data were analyzed using the Statistical Package for the Social Sciences (SPSS), version 27. Descriptive statistics were displayed in tables. In addition to finding Pearson’s correlation coefficients, we conducted multiple linear regression analyses to identify potential predictors of anxiety and depression. Mediation analyses were also conducted.

Skewed distributions of the HARS were normalized using inverse values, fifth roots for the ZBI factor 2 and ZBI composite score, and Log 10 for all other variables while applying two-part models [47]. Two-part modeling also involved recoding zeros as system missing values before normalizing continuous variables. Dichotomous variables were coded as one for the presence of back pain, caregiver burden, anxiety, and depression and zero for the absence of back pain, burden of care, anxiety, and depression. Logistic regression was used to ascertain whether back pain and burden of care predicted the presence or absence of depression and anxiety. Multiple linear regression analysis helped us observe whether the presence or absence of chronic back pain and caregiver burden predicted the degree of anxiety and depression.

The internal consistency of the Patient Health Questionnaire (PHQ-9), Zarit Burden Interview (ZBI), Oswestry Disability Index (ODI), and the Hamilton Rating scale (HRS) were assessed. The composite reliability for the ODI (α = 0.81, $\overset{―}{r}\;=\;0.30$) and PHQ-9 (α = 0.84, $\overset{―}{r}\;=\;0.37$) was good, showing unidimensionality. However, the ZBI (α = 0.82, $\overset{―}{r}\;=\;0.17$) and the HRS (α = 0.84, $\overset{―}{r}\;=\;0.27$) indicated multiple factors. A principal component analysis with orthogonal rotation assessed the unidimensionality of both variables (ZBI and HRS). Rotation component parallel analysis by log and slugger table determined two components. Component loadings > 0.4 were used to retain items. Items that reduced Cronbach’s alpha were removed (reliability analysis).

A p-value of less than 0.05 at 95% confidence intervals was used to determine significant thresholds for all tests.

## Results

### Participant characteristics

A summary of the participants’ demographic characteristics is in Table 1. Close to 200 caregivers (*n* = 184) of children with various chronic conditions participated in the study. The following are chronic health conditions with which the participants’ children were diagnosed: epilepsy, chronic kidney disease, congenital heart disease, and sickle cell disease. Most of the participants were female (**n* *= 154; 84%), with roughly 80% being mothers *(n* = 147). Additionally, the average age of female participants was 37 years-old (SD = 8%). In general, participants had at least three other children who were siblings of the sick child (31%). Almost half had tertiary education (43.5%), more than half had post-primary schooling (88.6%), and more than half had help with caregiving (83.2%). The median age of the children was 2 years. Nearly 90% earned less than 80,000 Nigerian naira, and over 90% were married.

**Table 1:** Demographic Characteristics.

Variable	N	%	Mean	*SD
Adult Age	---	---	37	8
Adult Gender: Male	30	16%		
Adult Gender: Female	154	84%		
Help with caregiving: Yes	153	83%		
Help with caregiving: No	31	17%		
Family History of Chronic Illness: Yes	16	9%		
Family History of Chronic Illness: No	168	91%		
*Income: less than 20,000	11	6%		
*Income: 20,000–40,000	29	16%		
*Income: 41,000–60,000	11	6%		
*Income: 61,000–80,000	9	5%		
*Income: more than 80,000	19	10%		
Child Age	---	---	4	2
Child Gender: Male	119	65%		
Child Gender: Female	65	35%		
Relationship to Caregiver: Mother	147	80%		
Relationship to Caregiver: Other	37	20%		
Parent Education Level: Primary	17	9%		
Parent Education Level: Secondary	62	34%		
Parent Education Level: Tertiary	80	44%		
Parent Education Level: Postgraduate	21	11%		

*SD means standard deviation.

*All income is in Nigerian Naira.

### Correlations

The burden of care factor 1 represented themes relating to *emotional response to caring* while the burden of care factor 2 represented themes relating to *physical and social life effects on carer*. There was an association between burden of care (total score) and anxiety (total score; *r* = 0.36; *p* < 0.01) as well as anxiety factor 1 (*r* = 0.36; *p* < 0.01) and anxiety factor 2 (*r* = 0.20; **p <* *0.01*)*. There was also a link between burden of care (total score) and depression (*r* = 0.45; *p* < 0.01), burden of care (*emotional response to caring*; *r* = 0.84; *p* < 0.01), and burden of care factor 2 (*r* = 0.74; *p* < 0.01). Additionally, there was a correlation between burden of care (*emotional response to caring)* and adult gender (*r* = 0.17; *p* < 0.05), child/ caregiver relationship (**r* *= -0.19; *p* < 0.05), depression (*r* = 0.57; *p* < 0.01), anxiety factor 1 (*r* = 0.42; *p* < 0.01), anxiety factor 2 (*r* = 0.19; *p* < 0.05), anxiety (total score; *r* = 0.41; *p* < 0.01) and burden of care (**physical and social life effects on carer; r* *= 0.27; *p* < 0.01).

There was a relationship between back pain and anxiety (total score; *r* = 0.35; *p* < 0.01) as well as anxiety factor 1 (*r* = 0.27; *p* < 0.01) and anxiety factor 2 (*r* = 0.42; **p <* *0.01*)*. There was also an association between back pain and depression (*r* = 0.20; *p* < 0.01), number of children the caregiver has (*r* = 0.27; *p* < 0.01), and secondary level of education (*r* = 0.19; *p* < 0.01). There was an association between anxiety (total score) and the number of children a caregiver has (*r* = 0.22; *p* < 0.01), child/ caregiver relationship (*r* = -0.15; *p* < 0.05), depression (*r* = 0.73; *p* < 0.01), anxiety factor 1 (*r* = 0.95; *p* < 0.01), and anxiety factor 2 (*r* = 0.72; *p* < 0.01). There was an association between depression and anxiety (total score; *r* = 0.73; *p* < 0.01), anxiety factor 1 (*r* = 0.77; *p* < 0.01), anxiety factor 2 (*r* = 0.31; *p* < 0.01), and child/ caregiver relationship (*r* = -0.16; *p* < 0.05). Table 2. shows the correlation among sociodemographic factors and correlates of anxiety, depression, and chronic back pain.

**Table 2:** Correlation Matrix.

Variable name	PHQ-9	HARS Total	HARS Factor 1	HARS Factor 2	ODI Total	Zarit Total	Zarit Factor 1	Zarit Factor 2
Relationship to Caregiver	**-0.162** ^*^	**-0.145** ^*^	**-0.163** ^*^	-0.081	0.057	-0.127	**-0.188** ^*^	0.023
Postgraduate	0.022	0.011	0.004	0.035	-0.121	-0.019	-0.016	-0.012
Tertiary Education	-0.135	-0.142	-0.160^*^	-0.022	-0.100	-0.030	-0.054	0.022
Secondary Education	0.135	0.137	0.137	0.047	**0.193** ^**^	0.068	0.070	0.025
Primary Education	0.000	-0.003	0.023	-0.049	0.016	0.007	0.018	-0.011
No of children caregiver has	0.138	**0.217** ^**^	**0.228** ^**^	**0.161** ^*^	**0.270** ^**^	0.047	0.047	0.025
No of children living with caregiver	-0.041	0.001	-0.002	0.028	0.128	0.032	0.009	0.055
Family History of Chronic illness	-0.002	-0.046	-0.027	-0.067	0.033	-0.031	-0.025	-0.020
Help with caregiving	-0.009	-0.003	0.040	-0.111	-0.019	0.044	-0.046	0.124
Gender (C)	-0.007	-0.003	-0.009	0.013	0.037	-0.069	-0.022	-0.065
Income	-0.135	-0.157	-0.165	-0.109	-0.209	-0.083	0.082	-0.173
Gender (A)	-0.112	-0.090	-0.118	-0.008	0.093	-0.125	**-0.171** ^*^	-0.003
Age (A)	0.035	0.080	0.087	0.099	0.042	0.009	0.003	0.001

* Correlation is significant at the 0.05 level (2-tailed).

** Correlation is significant at the 0.01 level (2-tailed).

Note: PHQ-9- Patient Health Questionnaire, HARS Total- Hamilton Anxiety Rating Scale Total score, ODI – Oswestry Disability Index, Gender (A)- Adult gender, Gender (C)- Child gender, Age (A)- Adult age, No- Number.

### Principal component analysis

We conducted a principal component analysis (PCA) with orthogonal rotation to assess the degree of one-dimensionality between the factors from the ZBI and HRS. Rotation component parallel analysis by log and slugger table determined two components for both variables. Component loadings > 0.4 were used to retain items. Items that reduced Cronbach’s alpha were removed (reliability analysis). The resulting ZBI retained 20 items, and HRS retained 12 items. The first component- Zarit factor 1(α = 0.85, R = 0.30) had good composite reliability. Zarit Component 1 retained 13 items with themes related to *emotional response to caring*. Zarit Component 2 (α = 0.71, **R* *= 0.3) had good composite reliability and retained 7 items with themes related to *physical, and social life effects on the carer*. The Cronbach’s alpha exceeded 0.7 for both subscales, and average inter-item reliability exceeded 0.3, indicating good internal consistency. The results of the PCA assessment are presented in Table 3.

**Table 3:** Rotated Component Matrix for Zarit Burden Interview (ZBI).

ZBI items	Emotional Response to Providing Care	Physical and Social Life Effects on Caregiver
Do you feel stressed between caring for your relative and trying to meet other responsibilities for your family or work?	0.22	**0.41**
Do you feel embarrassed by your relative’s behavior?	**0.55**	0.13
Do you feel angry when you are around your relative?	**0.60**	0.22
Do you feel that your relative currently affects your relationship with other family members or friends in a negative way?	**0.53**	-0.13
Are you afraid what the future holds for your relative?	**0.63**	0.03
Do you feel strained when you are around your relative?	**0.59**	0.06
Do you feel that you do not have as much privacy as you would like because of your relative?	**0.53**	0.07
Do you feel that your social life has suffered because you are caring for your relative?	-0.12	**0.59**
Do you feel uncomfortable about having friends over because of your relative?	**0.72**	0.07
Do you feel that you have lost control of your life since your relative’s illness?	**0.77**	0.11
Do you wish you could just leave the care of your relative to someone else?	**0.68**	0.20
Do you feel uncertain about what to do about your relative?	**0.74**	0.06
Do you feel that you should be doing more for your relative?	**0.51**	-0.03
Do you feel you could do a better job in caring for your relative?	0.16	**0.44**
Overall, how burdened do you feel in caring for your relative?	0.21	**0.46**
Do you feel that your relative asks for more help than (s)he needs?	0.31	0.15
Do you feel that because of the time you spend with your relative that you do not have enough time for yourself?	**0.76**	0.15
Do you feel your relative is dependent upon you?	0.38	
Do you feel your health has suffered because of your involvement with your relative?	-0.17	**0.45**
Do you feel that your relative seems to expect you to take care of him/her as if you were the only one, he/she could depend on?	-0.05	**0.86**
Do you feel that you will be unable to take care of your relative much longer?	**0.53**	**0.85**

**Bold numbers** indicate component loadings > 0.4.

### Multiple regression analyses

#### Depression.

When exploring the degree in which burden of care (both subtests’ scores and overall score) and back pain were predictors of depression, results indicated that the total burden of care score predicted higher levels of depression (*b* = 0.53, *t* = 5.12, *p* = < 0.01, 95% CI = 0.32, 0.74) (see Tables 4 and 5).

**Table 4:** Multiple Regression Results for Independent Variables and Depression.

				95% CI for β	
	*Β*	β	P-value	Lower	Upper
**Model 1**					
Back Pain	0.26	0.22	0.06	-0.017	0.53
^a^Burden of care (emotional response)	0.53	0.61	**<0.001**	0.32	0.74
**Model 2**					
Back Pain	0.24	0.23	0.12	-0.06	0.55
^b^Burden of care (physical and social)	0.32	0.17	0.24	-0.22	0.85
**Model 3**					
Back Pain	0.18	0.18	0.14	-0.06	0.43
^c^Burden of care (Total score)	0.69	0.49	**<0.001**	0.36	1.03

Note:

a – refers to emotional response to providing care.

b – refers to physical and social life effects on caregiver.

c - indicates the sum of 22 items in (ZBI).

**P value** - < 0.05 is significant.

**Table 5:** Multiple Regression Results for Independent Variables and Anxiety.

				95% CI for β	
	*Β*	β	P-value	Lower	Upper
**Model 1**					
Back Pain	0.19	0.18	0.28	-0.16	0.55
^a^Burden of care (emotional response)	0.16	0.17	0.28	-0.13	0.44
**Model 2**					
Back Pain	0.43	0.38	**0.01**	0.12	0.75
^b^Burden of care (physical and social life)	-0.22	-0.11	0.45	-0.79	0.36
**Model 3**					
Back Pain	0.28	0.26	0.06	-0.01	0.57
^c^Burden of care (Total score)	0.56	0.32	**0.02**	0.09	1.03

Note:

a – refers to emotional response to providing care.

b – refers to physical and social life effects on caregiver.

c - indicates the sum of 22 items in (ZBI).

**P value** - < 0.05 is significant.

### Anxiety

Those same factors, burden of care and back pain scores, were also analyzed using multiple regression. Results showed that the burden of care (including scores for the two subtests and overall test) and back pain on back pain, indicated that the burden of care predicted higher levels of anxiety (*b* = 0.56, *t* = 2.44. *p* = 0.02, β = 0.32, 95% CI = 0.09, 1.03) in the final regression model.

### Mediation analysis

#### Burden of care on depression by chronic back pain.

**Fig 1:**
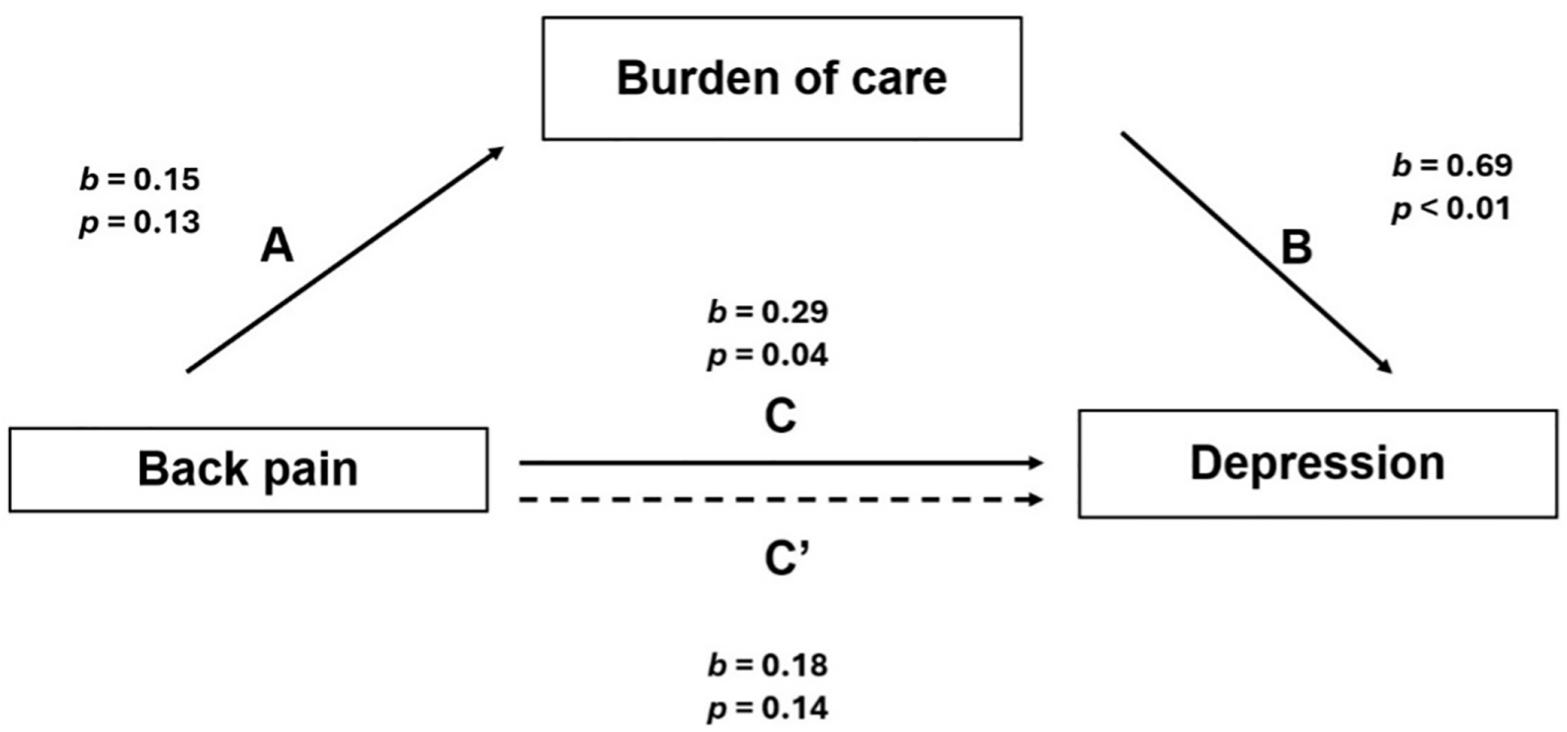
Mediation model for burden of care on back pain and depression.

Fig 1 depicts the results from the mediation analysis, specifically with the burden of care on back pain and depression, that we conducted. We initially thought that the caregiver’s burden of care could meditate the relationship between back pain and depression; however, results indicated that it was not a mediator for that relationship. Results also indicated that back pain was a predictor for depression among caregivers (*b* = 0.29, *p* = 0.04, *t* = 2.11, [95% CI = 0.15, 0.56]), which was consistent with our initial hypothesis. Path A coefficients (back pain to the burden of care) was not significant (**b* *= 0.15, **p* *= 0.13, *t* = 1.54, [95% CI = - 0.05, 0.35]), which means that back pain did not predict the burden of care. However, the Path B coefficient (burden of care to depression) was significant (*b* = 0.69, *p*** <** 0.01, *t* = 4.14, [95% CI = 0.36, 1.03]); a resul*t* that was also consistent with our initial hypothesis. Path C coefficients (back pain to the burden of care and depression) were significant (*b* = 0.29, *p* = 0.04, *t* = 2.11, [95% CI = 0.15, 0.56]), which was consis*t*ent with our second hypothesis.

While results from this analysis showed positive indirect effects (*b* = 0.104, [95% CI = - 0.02, 0.23]) that were lower than that of the direct effect (*b* = 0.18, *p* = 0.14, *t* = 1.5, [95% CI = - 0.06, 0.43]), no mediation occurred, as the difference between the indirect and direct coefficients was not significant. Additionally, results further indicated a potential power challenge despite there being positive total and indirect effects.

#### Burden of care on anxiety by chronic back pain.

**Fig 2:**
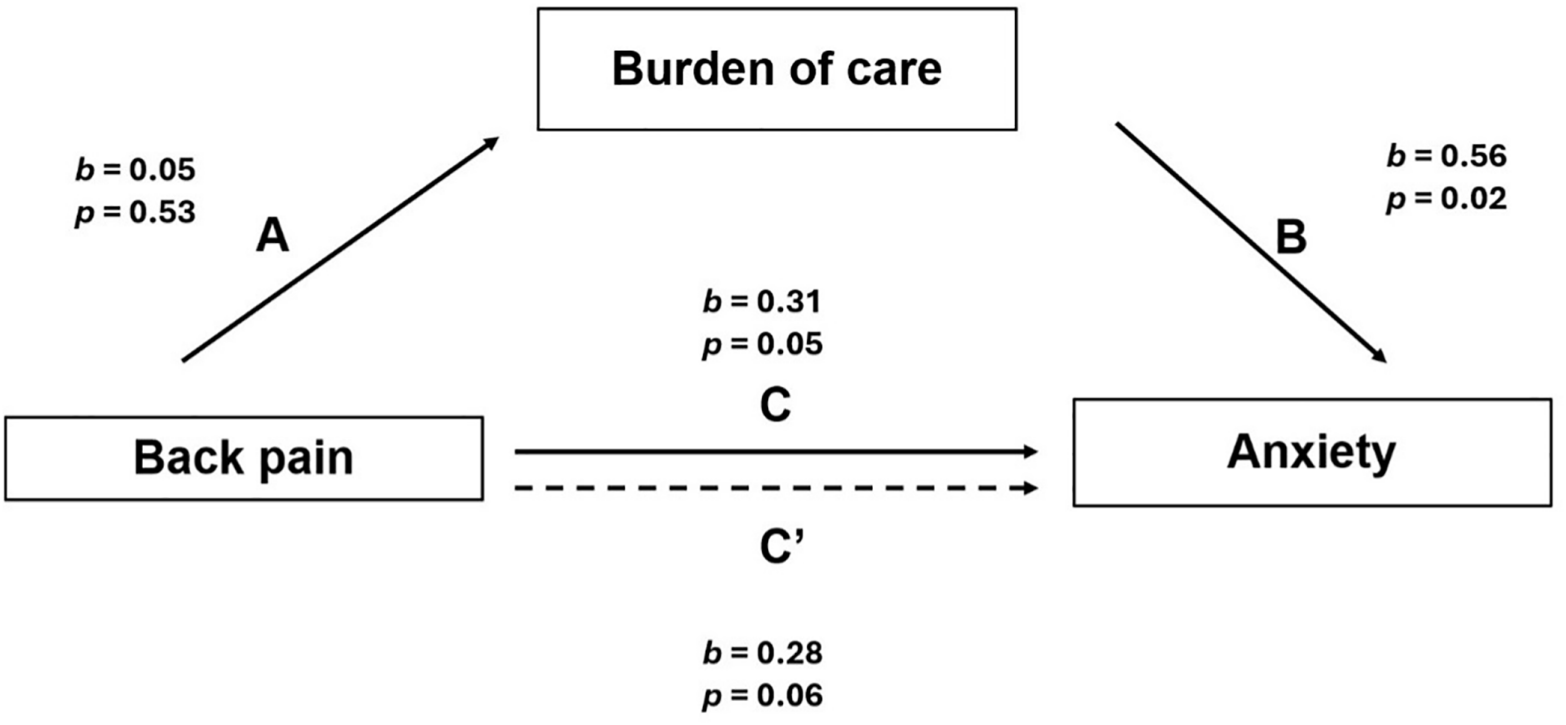
Mediation model for burden of care on back pain and anxiety.

Fig 2 depicts the mediation analysis of the care burden on back pain and anxiety. Results indicated that the burden of care did not mediate the relationship between back pain and anxiety, which contradicted our initial expectation. However, results also showed that back pain predicted anxiety as evidenced by significant total effects (*b* = 0.31, *p* = 0.05, *t* = 2.01, [95% CI = 0.006, 0.62]). Path A coefficients (back pain to the burden of care) was not significant (b = 0.05, *p* = 0.53, t = 0.63, [95% CI = - 0.12, 0.24]), meaning, back pain was not a predictor for caregiver’s burden of care. Path B coefficients (burden of care to anxiety) was significant (*b* = 0.56, *p* < 0.02, *t* = 2.4, [95% CI = 0.09, 1.03]); which was also consis*t*ent with our initial hypothesis. Path C coefficients (back pain to the care burden and depression) were significant (*b* = 0.31, *p* = 0.05, *t* = 2.01, [95% CI = 0.006, 0.62]), which was consis*t*ent with our second hypothesis.

Results also showed positive indirect effects (*b* = 0.03, [95% CI = - 0.076, 0.15]), the *b* value was lower than that of the direct effect (*b* = 0.28, *p* = 0.06, *t* = 1.93, [95% CI = - 0.01, 0.57]); however, no mediation occurred. These findings further indicate a possible power challenge given there was no evidence of mediation and the difference between indirect and direct effects was not significant.

## Discussion

The purpose of the study was to explore the prevalence and relationship between anxiety, depression, and chronic back pain among caregivers of children with chronic health conditions in Nigerian tertiary hospitals. We also explored the degree to which these factors were associated with their burden of care. Significant findings from the study showed a high prevalence of anxiety, chronic back pain, and depression among caregivers of children with chronic conditions. We also found that higher levels of burden of care were associated with anxiety and depression among caregivers of children with chronic conditions. The burden of care did not mediate the relationship between chronic back pain and depression and anxiety.

In line with the results of this study, several studies have documented a high prevalence of anxiety and depression among caregivers of children with chronic conditions [7,8,48]. Most caregivers, predominantly mothers, reported a higher burden of care, like in other studies [49–51]. Although some studies have reported higher levels of caregiver burden among fathers [1,52], another study reported no difference in caregiver burden between fathers and mothers [2]. This may be due to prevailing cultural factors that encourage fathers to be more available as caregivers and availability of social support in the case of indifference to caregiver burden and poor social, physical, and financial resources in the former.

We also found that chronic back pain was prevalent among the caregivers of chronic conditions in this study, which is consistent with previous studies that linked caregiving with increased physical demands resulting from the development of musculoskeletal problems [13,53–56]. This finding underscores that caregivers are more likely to experience the physical strain due to lack of necessary infrastructure, resources, and the significant demands of caring for chronically ill children.

Multiple regression results showed that chronic back pain was a predictor for anxiety and depression, which is consistent with previous research that examined the relationship among those same variables [57,58]. Caregivers of children with chronic illnesses usually sit for long hours by their child’s hospital beds, in addition to pushing them in wheelchairs (temporarily or permanently), and carrying heavy equipment utilized in providing care for sick children [59–61]. These activities contribute to caregivers experiencing sprained back muscles, muscle spasms, and wear- and-tear of spine ligaments, all of which may result in chronic back pain [59].

There is a bidirectional relationship between physical pain and psychological distress, including anxiety and depression, explained in part by a shared neural mechanism [62]. This finding suggests that frequent comorbidities or shared risks may exist among these conditions [62]. In individuals with back pain, functional imaging has demonstrated dysfunction in neural networks that process emotional stimuli, especially in the anterior cingulate cortex and the prefrontal cortex [63,64]. Aberrant activity in the insula, the region associated with the processing of painful stimuli, has been observed in individuals diagnosed with depression [65].

Similar activation patterns have been observed in individuals with chronic pain and anxiety, namely in the thalamus, prefrontal cortex, and anterior cingulate cortex. Furthermore, unpleasant sensations associated with the physical pain caregivers experience may result in the activation of areas of the brain in those who are predisposed to anxiety and depression. Anxiety and depression can also increase the perception of pain by lowering the pain threshold and leading to less inhibition of pain in the descending pain pathways. The interplay between emotional and physical pain is often mediated by endorphins and enkephalins, both of which, function as neurotransmitters in pain modulation. These peptides bind to opioid receptors in the central and peripheral nervous systems, influencing both physical pain perception and emotional states. Research indicates that dysfunctions in these opioid systems may contribute to chronic pain conditions and emotional distress [66]. Patients with chronic pain with comorbid depression and anxiety often have poorer treatment outcomes [67]. Therefore, it is important to screen for and adequately treat chronic back pain, anxiety and depression among these populations to enhance their quality of life and general well-being.

In the mediation analyses, the burden of care did not mediate the association between back pain and anxiety or depression. However, there was a direct relationship between the burden of care and depression and between back pain and depression. Similar results were found for the mediation analyses for anxiety. Therefore, the relationship between back pain and either anxiety or depression is independent of the burden of caring for patients with chronic illnesses. Factors that have been reported to mediate the relationship between chronic pain include pain catastrophizing, pain efficacy, family resilience, and good family communication and problem-solving [68]. These factors were not explored in this study.

Various limitations should be considered when reporting these findings. First, the study’s cross-sectional design or layout does not allow for a temporal relationship between chronic pain, anxiety, and depression. Second, the study was conducted in tertiary hospitals and may not be generalizable to caregivers in other settings, given the difference in patient and caregiver profiles in primary and secondary healthcare settings. Third, the exclusion of patients with intellectual disabilities and psychiatric illnesses also limits the generalizability of the study. Fourth, the study employed convenience sampling, which may have introduced selection bias into the study and may have caused a non-representative sample of the population of interest. Fifth, the comparatively small sample size may introduce bias in the estimates, either inflating or attenuating them. The results should be interpreted cautiously, and further research with larger samples is recommended.

In conclusion, this study is among the few to examine the interconnectedness of chronic back pain, anxiety, and depression in caregivers of children with chronic medical conditions in Nigeria. It revealed a high prevalence of these conditions and established that caregiver burden is significantly associated with increased levels of anxiety and depression. Notably, the burden of care did not mediate the relationship between chronic back pain and psychological distress, suggesting that other factors may contribute to this link. These findings advance knowledge in the caregiving literature and contribute to the Sustainable Development Goals (SDG), specifically SDG 3 (Good Health and Well-being) and SDG 5 (Gender Equality) by drawing attention to the physical and emotional toll of caregiving, particularly on women. Future research should explore alternative mediators such as family resilience and pain efficacy, using longitudinal methods to better understand these complex dynamics. Interventions should include regular mental health and physical health screenings for caregivers, targeted support systems, and redesign of hospital environments to reduce caregiver strain. Policymakers and health system leaders must recognize the hidden burden caregivers carry and commit to integrated strategies that support both caregiver and patient well-being.

## Supporting information

S1 Data SPSS Final cleaned data. (ZIP)

S1 Checklist Strobe Checklist. (DOC)

S2 Checklist Inclusivity in Research Questionnaire. (DOCX)
